# What Fuels Natural Killers? Metabolism and NK Cell Responses

**DOI:** 10.3389/fimmu.2017.00367

**Published:** 2017-04-03

**Authors:** Clair M. Gardiner, David K. Finlay

**Affiliations:** ^1^School of Biochemistry and Immunology, Trinity Biomedical Sciences Institute, Trinity College Dublin, Dublin, Ireland; ^2^School of Pharmacy and Pharmaceutical Sciences, Trinity Biomedical Sciences Institute, Trinity College Dublin, Dublin, Ireland

**Keywords:** natural killer cells, metabolism, mTORC1 signalling, glycolysis and oxidative phosphorylation, innate immune response, adaptive immune response, glucose, viral infection, tumor immunotherapy

## Abstract

There is a growing appreciation that cellular metabolism is important in determining the course of lymphocyte responses. Additionally, changes in metabolic processes have been linked to dysfunctional lymphocyte functions in a number of different diseases. While most early studies of metabolic regulation of lymphocyte function focused on T lymphocytes, an understanding of how metabolic pathways impact upon natural killer (NK) cell responses is now starting to emerge. In this review article, we will discuss how cellular metabolism influences lymphocyte function with a particular focus upon NK cells.

## Introduction

Natural killer (NK) cells are important effector lymphocytes that are best characterized for their antiviral and anticancer activities (1, 2). They carry out direct cytotoxicity of target cells and are potent producers of IFNγ cytokine. In addition to their effector functions, NK cells can also regulate development of the adaptive immune response in a variety of ways including production of immune modulating cytokines and regulating DC maturation (3). As NK cell responses can be detected very rapidly after infection (within hours), they have historically been classified as cells of the innate immune system. However, this textbook viewpoint has changed with the discovery of long-lived and sustained functional NK cells, and the demonstration of intrinsic innate immune memory (4–6).

Natural killer cells are also clinically important and represent a good target for anticancer immune therapy in which the host immune system is harnessed for anticancer activities. Currently, NK cells are being investigated in clinical trials using a range of different approaches. These include direct activation *in vivo* by blocking various inhibitory NK cell receptors using antibodies against KIR and NKG2A (see www.innate-pharma.com). Recently, one such trial investigating an anti-KIR antibody as a single agent for the treatment of acute myelogenous leukemia (AML) did not reach its primary efficacy endpoint and was halted. However, given the promising results in preclinical studies (7), it will be interesting to see the results of several other trials still on-going that use these antibodies in combination with other agents for a range of cancer types.

Natural killer cells (allogeneic, haploidentical) are also successfully being used for adoptive transfer treatment of AML (8–10). Adoptive transfer therapy allows the potential to genetically manipulate NK cells prior to infusion. This concept is being explored in a number of clinical trials (NCT01974479 and NCT00995137) that have generated chimeric antigen receptor (CAR) NK cells, designed to recognize and treat B cell acute lymphoblastic leukemic. While these trials are using primary NK cells, there is also some evidence that CAR-modified NK cell lines (NK-92) can provide benefit in different preclinical models (11, 12). Finally, NK cells are important in particular antibody-mediated immunotherapy settings, for instance for the treatment of neuroblastoma or lymphoma where they mediate antibody-dependent cellular cytotoxicity (ADCC) against tumor cells (13). Understanding the relevance of metabolism to NK cell effector functions will provide new mechanisms to enhance these therapeutic approaches but also opens up the potential for new avenues of NK cell-based therapies as discussed below.

## Metabolism and Lymphocyte Responses

It is becoming clear that metabolism is profoundly important for immune function, to the extent that manipulation of metabolism can alter immune cell fate and function. Immune responses involve highly dynamic changes in immune cell function, which often encompass robust cellular growth and proliferation. Therefore, it is not surprising that there are corresponding changes in metabolism that match the dynamic nature of immune cells.

**Figure 1:**
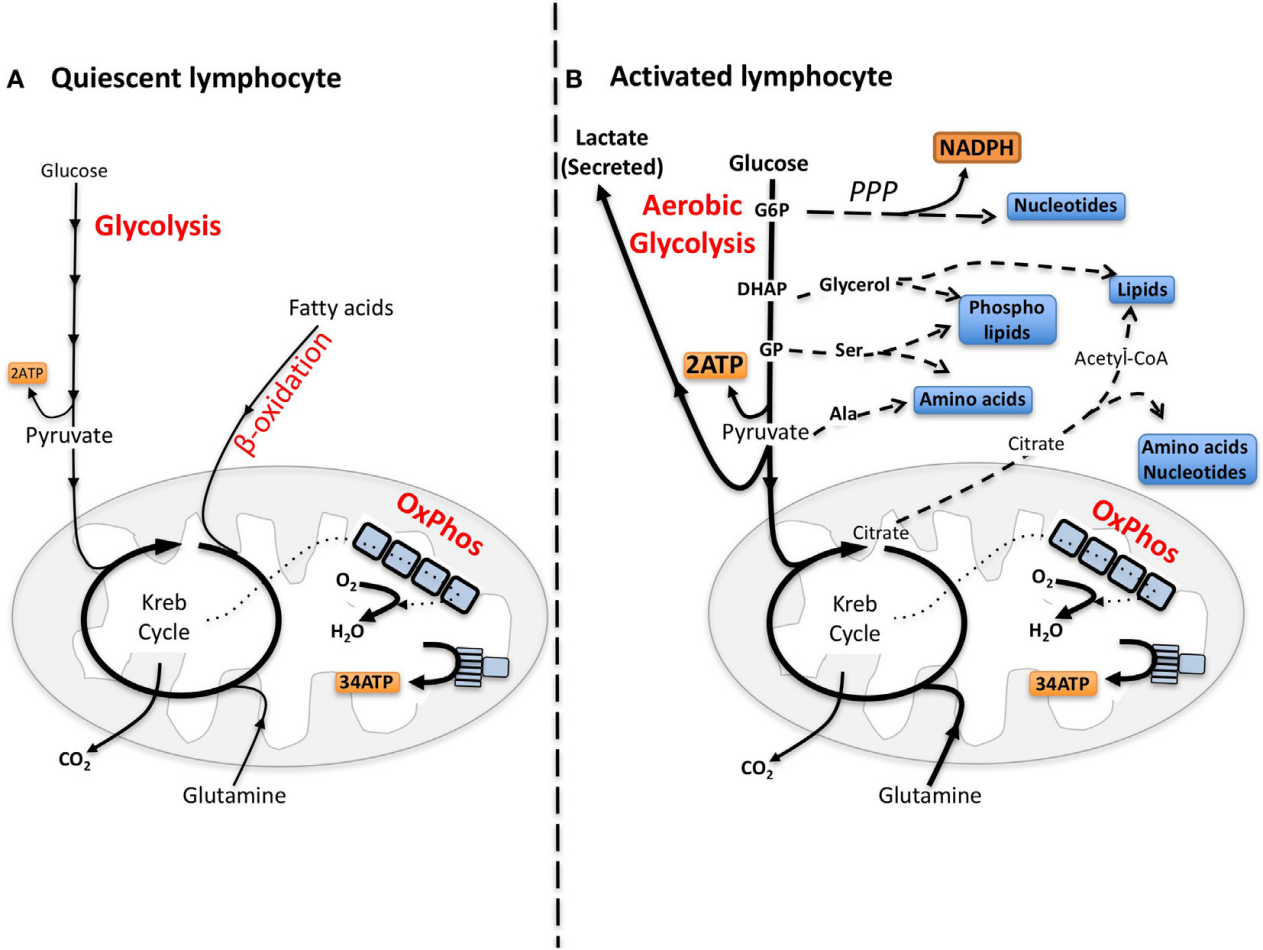
**The differing metabolic phenotypes of quiescent versus activated lymphocytes**. **(A)** Adenosine triphosphate (ATP) is the key molecule that provides energy for cellular processes. Maintaining cellular ATP levels is essential for bioenergetic homeostasis and cell survival. Glucose, a key fuel source for mammalian cells, can be metabolized *via* two integrated metabolic pathways, glycolysis and oxidative phosphorylation (oxphos), that efficiently generate ATP. Glycolysis converts glucose to pyruvate that, following transportation into the mitochondria, is further metabolized to CO_2_ by the Krebs cycle fueling oxphos and ATP synthesis. In addition to the breakdown of glucose *via* glycolysis, cells have the ability to metabolize alternative substrates including fatty acids by β-oxidation and glutamine by glutaminolysis, which feed into the Krebs cycle and drive oxphos. **(B)** Aerobic glycolysis supports biosynthetic processes of the cell as it allows the uptake of larger amounts of glucose and the maintenance of elevated glycolytic flux. Glycolytic intermediates are then diverted into various pathways for the synthesis of biomolecules that support biosynthetic processes. For instance, glucose-6-phosphate (G6P) generated by the first step in glycolysis can feed into the pentose phosphate pathway (PPP) to support nucleotide synthesis. This pathway also generates NADPH, a cofactor that is essential for various biosynthetic processes including lipid synthesis. Glucose can also be converted into cytoplasmic acetyl-CoA *via* citrate in the Krebs cycle for the production of cholesterol and fatty acids for lipid synthesis. Other glycolytic intermediates can also be converted into biomolecules used for protein and lipid synthesis. During aerobic glycolysis a significant proportion of pyruvate is also converted to lactate and secreted from the cell. Lactate export is important as it allows glycolysis to be sustained at an elevated rate. Alternative fuels including glutamine feed into the Krebs cycle and can also supply biomolecules for biosynthetic processes under certain conditions. DHAP, dihydroxyacetone phosphate, GP, glycerate 3-phosphate, Ser, serine; Ala, alanine.

Quiescent lymphocytes have limited biosynthetic demands and metabolic pathways are tuned toward efficiently metabolizing glucose through glycolysis coupled to oxidative phosphorylation (oxphos) to make energy, i.e., adenosine triphosphate (ATP) (Figure 1). Upon immune activation, lymphocytes, including NK cells, increase glucose metabolism through glycolysis metabolizing much of the glucose into lactate, which is secreted from the cell, a process called “aerobic glycolysis” (14–17). Aerobic glycolysis is adopted by cells engaging in robust growth and proliferation because it provides the biosynthetic precursors that are essential for the synthesis of nucleotides, amino acids, and lipids (Figure 1) (18, 19). Therefore, for cells engaged in aerobic glycolysis, the primary function of glucose has shifted from a fuel to generate energy to a source of carbon that can be used for biosynthetic purposes (18).

Beyond the biochemistry of energy production and cellular biosynthesis, it has emerged that metabolism also plays a direct role in controlling immune signaling and immune cell effector functions (20). For instance, the glycolytic enzyme GAPDH also controls the expression of immunological molecules in both myeloid cells and lymphocytes through binding to the 3′UTR of mRNA (21, 22). In addition, certain metabolic intermediates have emerged as important signaling molecules, e.g., the glycolytic intermediate phosphoenolpyruvate regulates Ca^2+^ signaling and nuclear factor of activated T cells activity during T cell activation (23).

## NK Cell Metabolism

### Murine NK Cells

In mice, NK cells develop and mature in the bone marrow and expression levels of CD11b and CD27 can be used to define subset progression to terminally differentiated naïve NK cells. Immature cells had higher expression of nutrient receptors CD71 (transferrin receptor) and CD98 (l-amino acid transporter), and higher uptake of 2-NBDG (a fluorescent glucose analog) presumably to facilitate the metabolic demands of proliferation and differentiation (16). All these measurements reduced as cells matured such that resting naïve NK cells had a phenotype that was more characteristic of a quiescent lymphocyte. This was also reflected in transcriptional profiles of metabolic genes analyzed with naïve cells enriched for genes associated with oxphos, fatty acid oxidation (FAO), and autophagy compared with proliferating progenitor cells (16). Keppel et al. had similar findings in that freshly isolated splenic NK cells had low levels of metabolic activity with a preference for oxphos rather than glycolysis (24). Short-term activation (4–6 h) with either cytokines or receptor ligation did not significantly alter the metabolic pathways used by NK cells (24). However, when NK cells are stimulated with cytokine over longer periods of time, as might be expected *in vivo* in response to infection, changes in NK cell metabolism become apparent. Activation of murine splenic NK cells for 3–5 days with high concentrations of IL15 (100 ng/ml) upregulated both glycolysis and oxphos (16, 24). Our data with NK cells expanded from splenocytes and primed with low dose IL15 demonstrated significant upregulation of both glycolysis and oxphos in response to IL2 + IL12 for 18 h. Although both pathways were upregulated, there was a preferential increase in glycolysis, a finding that parallels metabolic changes in CD8 effector T cells. Whether other fuels such as fatty acids and glutamine are important for NK cell metabolism and function is, to date, largely unexplored.

The mammalian target of rapamycin complex 1 (mTORC1) is considered a master regulator of immunology and metabolism. Increasingly, the delineation between these activities blurs as the importance of metabolism on immune cell function is appreciated. mTORC1 was shown to be important for the development and maturation of murine NK cells and is strongly upregulated in mature NK cells in response to IL2 or IL15 (14, 16), cytokines that potently activate NK cell functions. mTORC1 activity is required for the increased glycolysis observed in NK cells stimulated for 18 h with IL2/IL12 or high dose IL15 (14, 17). The exact mechanisms by which mTORC1 controls NK cell metabolism have not yet been described, though based on studies in T lymphocytes the transcription factors HIF1α or cMyc are likely to be involved (20). mTORC2 signaling through the kinases Akt and SGK1 has recognised roles in regulating the differentiation of T cell subsets (25–27). Interestingly, while Akt regulates cellular metabolism in various cell types, this is not the case in IL2-maintained CD8 cytotoxic T cells (26). A role for mTORC2 in NK cell metabolism and function remains to be investigated.

It is clear that NK cells alter their metabolism in tandem with acquisition of enhanced effector functions but this does not necessarily imply a direct relationship between the two. A range of experimental approaches have been used to interfere with particular metabolic pathways to test the impact on NK cell function. These approaches demonstrated that direct metabolic restriction of glycolysis results in decreased NK cell effector function (14, 24).

Natural killer cells are part of a wider family of innate lymphoid cells (ILCs) and to date nothing is known about how cellular metabolism impacts upon the other ILC subsets. However, one study has highlighted a role for mTORC1 for cytokine production in type 2 ILCs, which might suggest that metabolic changes are involved (28).

### Human NK Cells

While less is known about human NK cell metabolism, it is likely that distinct phenotypic and functional NK cell subsets will have characteristic metabolic signatures. CD56 expression levels have long been used to define peripheral blood NK cells subsets in humans. CD56^dim^ NK cells account for approximately 90% of circulating NK cells (2). These cells express CD16 and KIR and are more cytotoxic. By contrast, CD56^bright^ cells predominate in tissues. They are less good at killing (granzyme B expression must be induced by cytokine) but are more potent at IFNγ production. Although there is strong evidence demonstrating that CD56^bright^ cells can differentiate into CD56^dim^ cells, both populations are maintained at a steady state in the peripheral blood of normal healthy individuals, suggesting that CD56^brights^ are not merely a precursor in a developmental pathway and that they may have particular specialized functions, possibly related to tissue trafficking and immunosurveillance. We have recently described that CD56^bright^ and CD56^dim^ subsets can also be distinguished in terms of their metabolism (15).

mTORC1 is a key regulator of metabolic responses and multiple cytokines have been shown to induce mTORC1 activity in human NK cells (15, 16, 29). Interestingly, mTORC1 was much more strongly activated in CD56^bright^ cells and these cells responded more strongly to cytokine in terms of nutrient receptor expression and 2-NBDG uptake. This ensures that CD56^bright^ cells that produce large amounts of IFNγ can meet the associated increased biosynthetic and metabolic demands of their specialized functional activity. However, the exact role for mTORC1 in CD56^bright^ and CD56^dim^ NK cells requires further investigation as initial observations show mTORC1 is required for IL2-induced, but not IL12/IL15-induced, metabolic responses (15). Interestingly, in line with the observations that CD56^bright^ NK cells are more metabolically responsive to cytokine stimulation, metabolic restriction had a greater impact upon the function of CD56^bright^ versus CD56^dim^ NK cells (15).

More recently, within the CD56^dim^ subset, progress has been made in delineating adaptive (cells that have experienced HCMV) and canonical NK cells. Cell surface, intracellular markers, and epigenetic signatures have been defined and important functional differences have been defined (30, 31). It is likely that these subsets will be characterized by different metabolic pathway usage and based on similarities between adaptive NK cells and memory T cells, it seems reasonable to predict that adaptive NK cells may be characterized by preferential use of FAO metabolism to fuel oxphos (32). In mice, using Ly49H to identify cytomegalovirus (CMV)-experienced NK cells will also be informative in terms of metabolic analyses and data are already starting to emerge to support that NK cell metabolic responses will play a role in the formation of NK cell memory (see below). Finally, it will be interesting to investigate if there are differences between licensed and unlicensed NK cells in terms of their metabolism (33). If normal responsive NK cells, i.e., licensed, use mTORC1 to metabolically reprogramme and upregulate functions, we might predict that unlicensed NK cells which are functionally hyporesponsive may be deficient in terms of mTORC1 activation in response to cytokine, which would have a downstream impact on their metabolism and function.

## NK Cell Metabolism and Viral Infections

Most of what is known about metabolic changes in lymphocytes during viral infections come from studies on CD8 T cells and thusfar, very little is known about metabolic changes that occur in NK cells during viral infection. The underlying principles of CD8 T cells metabolism during viral infection are likely to be similar in NK cells, though there may be differences in particular NK cell subsets (33). The CD8 T cell response to virus consists of an initial expansion of virus-specific T cells that carry out effector functions against virally infected cells. During this process, metabolism is altered from utilization of oxphos (fueled by glucose and glutamine) and FAO in naïve T cells (34, 35) to increased glycolysis and oxphos in effector T cells (36–39). As expected, mitochondrial mass, ATP levels, and spare respiratory capacity (SRC) can all be increased in effector cells (39). mTORC1 is a key in terms of driving metabolic reprogramming of T cells (40, 41) and although much remains to be elucidated in terms of molecular mechanisms, PGC1α, a regulator of mitochondrial biogenesis has recently been implicated in a number of murine models (38, 39). After immune control has been established by elimination of the pathogen, the expanded effector population contracts to generate a small pool of long-lived memory cells that have the capacity to react quickly and more effectively upon subsequent exposure to the same virus. These memory cells preferentially use FAO to fuel oxphos to provide energy (32, 42). They also have increased mitochondrial mass and mitochondrial SRC (compared to naïve or effector T cells) which gives them a bioenergetics advantage for their increased effector functions upon activation.

However, sometimes the immune system fails to control the pathogen and chronic viral infections can ensue that are associated with increased long-term morbidity and mortality in patients, e.g., HIV-1 and hepatitis B or C infections. From an immunology perspective, lymphocytes in these patients develop an “exhausted” phenotype and although present, they are hyporesponsive. Metabolically, exhausted cells have impaired mitochondrial responses that paradoxically include increased mitochondrial mass but reduced oxphos, SRC, and mitochondrial membrane potential (38, 43). For at least LCMV infections, mitochondrial ROS is increased in parallel (38). There is a general consensus that defective mitochondrial function is a characteristic of chronic infection and that the reduced mitochondrial activity may force the cells to ineffectively utilize glycolysis under conditions where glucose availability is limited. Thus, while we do not fully understand the why and how, it is clear that different T cell subsets are characterized by using different metabolic pathways for their survival and function.

Although little is yet known about how metabolism in NK cells impacts upon viral infections, information is starting to emerge, albeit indirectly. There is a large body of literature supporting the importance of NK cells in the immune response to CMV with the demonstration that CMV can shape the NK cell repertoire and generate NK cells that have memory for CMV (6). While the in-depth metabolic changes in NK cells during CMV infection remain to be characterized, a sneak-preview is provided by O’Sullivan et al. that show reduced mitochondrial polarization and increased mtROS in effector NK cells (44). As these effector cells died back to generate a pool of memory cells in a mitophagy-dependent manner, mitochondrial potential in memory NK cells was restored, concomitant with reduced mtROS, suggesting a role for metabolic regulation of innate immune memory as has been seen for memory CD8 cells (45).

Furthermore, Marcais et al. have demonstrated that intrinsic mTORC1 plays an important role in the NK cell response to MCMV infection, including proliferation, expression of granzyme B, and IFNγ production (to some but not all stimuli) (16); however, the relative contribution of mTORC1 to metabolism versus direct effects on functions remains to be elucidated.

## Implications for Disease

Metabolism remains an unexplored landscape that holds great potential for therapeutic manipulation of NK cells in a variety of diseases including cancer and chronic viral infection. There are several potential ways in which metabolism of NK cells could be targeted to improve outcome for cancer patients.

It has been known for a long time that NK cell functions are impaired in many different forms of cancer (46–48). Indeed, various mouse models have shown that NK cells become exhausted during cancer (49, 50). In addition to no longer being effective against cancer, reduced NK activity also leaves patients susceptible to infections. Therefore, activation of NK cells *in vivo* is an attractive therapeutic strategy. We predict that NK cell metabolism in these patients will be severely compromised and restoring normal metabolism might be key to restoring function. Check-point blockade inhibitors including anti-PD1 and -PDL1 have revolutionized many cancer treatments. They function in part by restoring immune activity in exhausted T cells and a recent report suggests that restoring metabolism in T cells can contribute to this (38). There is some evidence that NK cells from multiple myeloma patients expressed PD1 and that blocking PD1 enhanced NK cell functions against tumor cells (51). Indeed, anti-PD1 blockade increased expression of genes associated with NK cell cytotoxicity during *in vivo* therapy for cancer (52). There is also evidence that anti-Tim3 restored NK cell function in NK cells from patients with metastatic melanoma (53). However, the impact of check-point inhibitors on NK cell functions during cancer treatment has not yet been extensively investigated. Understanding how NK cell metabolism is altered in cancer will provide valuable information regarding how best to restore metabolism and function in exhausted NK cells.

As mentioned earlier, NK cells are used therapeutically in a variety of settings including adoptive transfer therapy and antibody-mediated immunotherapy for cancer. Adoptive transfer of allogeneic haploidentical NK cells is currently used as a treatment for AML (54–56). One benefit of allogeneic NK cells is the ability to culture the cells *ex vivo*, e.g., in the presence of cytokine, to increase immune function. Understanding how metabolism contributes to NK cell function will provide the opportunity to strategically manipulate metabolism of NK cells, which will translate into more effective anticancer activity *in vivo*. Proof of principle for this comes from preclinical studies in which altering T cell metabolism improved cancer outcome in an adoptive cellular immunotherapy setting. In a seminal paper, it was demonstrated that mitochondrial architecture not only alters metabolism but also function (57). Exploiting this, IL2-treated T effector cells were pharmacologically manipulated with drugs that promoted mitochondrial fusion and increased oxphos efficiency *ex vivo*. As a result, these T cells that were adoptively transferred had increased longevity *in vivo* and improved overall survival in this lymphoma model (57). *Ex vivo* manipulation of cells for adoptive transfer has the advantage of being able to use drugs and cytokines that may be toxic for direct *in vivo* administration. As NK cells are already used clinically for adoptive transfer therapy, understanding and improving metabolism of NK cells for improved function and improved clinical responses is a realistic goal.

Natural killer cells are also important in antibody immunotherapy for a variety of cancers including breast cancer (anti-Her2), lymphoma (anti-CD20), and neuroblastoma (anti-GD2), reviewed in Ref. (13). Increasing ADCC of NK cells will be beneficial for these patients. Metabolism provides an opportunity to do this as, similar to what has been observed for T cells, it is likely that particular functional subsets, e.g., adaptive NK cells that have high ADCC (30, 31), will have a different metabolism compared to canonical NK cells. By understanding NK cell metabolism in these subsets, we have the potential to manipulate metabolism to increase ADCC.

It is important to note that many therapies that identify new treatments for cancer do so in isolation from the immune system and do not consider the potential negative impacts of particular treatments on the immune system, e.g., rapamycin, which inhibits mTORC1, has recently been proposed as a treatment for neuroblastoma (58). However, rapamycin is also likely to inhibit metabolic reprogramming in NK cells (for which mTORC1 is required) and may potentially impair functions that would be beneficial to the patient.

## Concluding Perspectives

**Figure 2:**
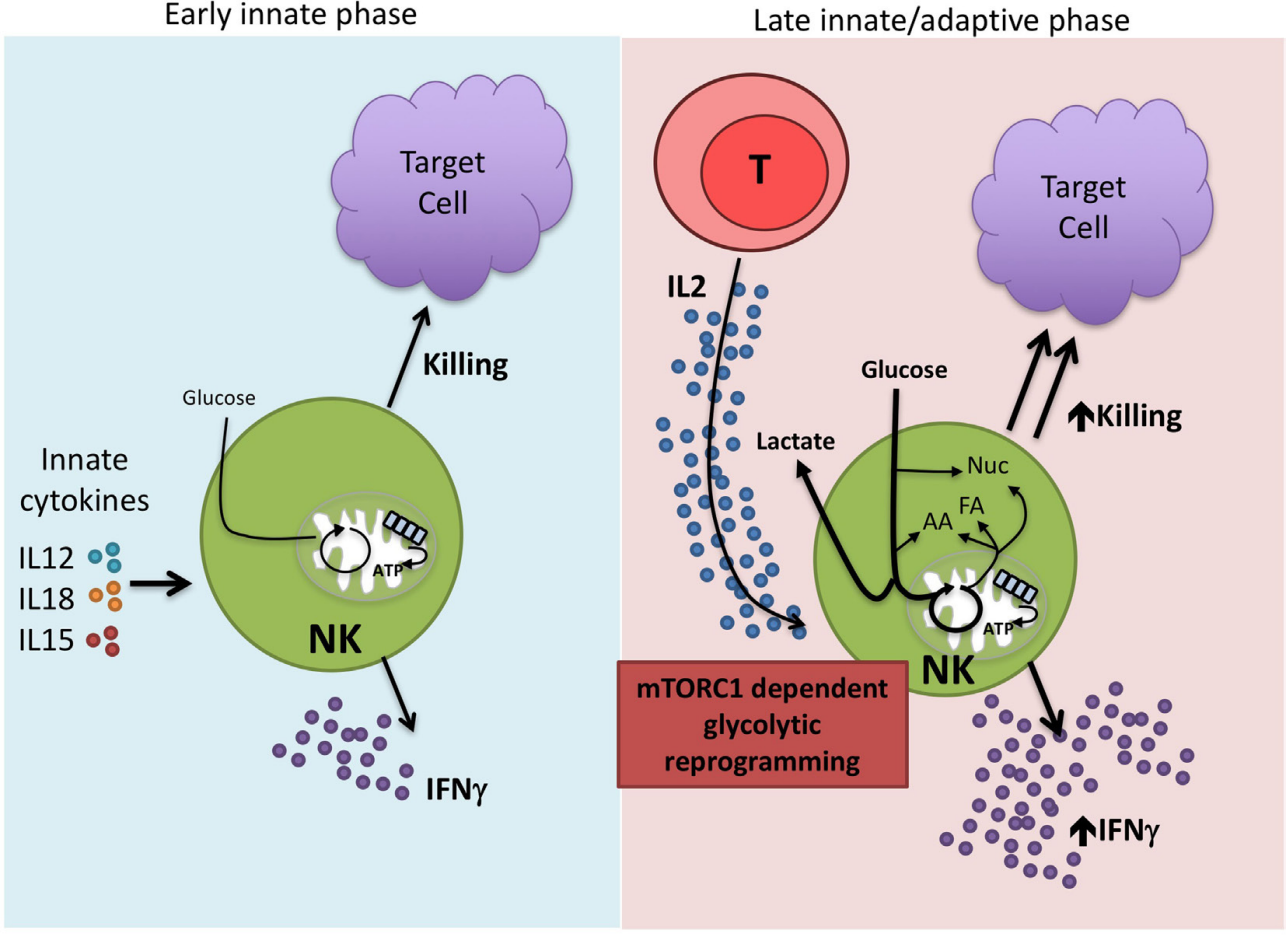
**Proposed bimodal model of natural killer (NK) cell activation**. NK cells are best characterized for their rapid, early immune responses after exposure to pathogen. Among the important innate cytokines that activate NK cells are IL12, IL15, and IL18. NK cells carry out direct cytotoxicity of target cells and are potent producers of IFNγ. There are relatively modest changes in NK cell metabolism at these early time points suggesting that this first wave of NK activity relies on cells that are primed and ready to go without the need for substantial metabolic reprogramming. However, we know that NK cells can function at extended time periods beyond the first few days post-infection. IL2, produced by activated T cells, is one cytokine that is important in this process. IL2 drives mTORC1-dependent glycolytic reprogramming of NK cells which we believe is critical for the sustained effector functions of this second generation of NK cells, allowing them to work in parallel with the adaptive immune response.

Overall, the data lead us to propose a bimodal model of NK cell immune function in terms of metabolic responses (Figure 2). Circulating NK cells respond rapidly upon activation by early innate cytokines and are important in the innate immune response. There are only modest changes in cellular metabolism associated with these activities and thus, metabolism is unlikely to impact significantly in regulating these functions. However, we know that NK cells have relatively low levels of CD25 and this becomes upregulated on NK cells during early activation to give a high-affinity IL2 receptor (59). This now enables the NK cell to respond to IL2 which is produced as part of the adaptive immune response. Thus, a second wave of NK cell activation commences to produce sustained NK cell activation and functioning in parallel and in concert with the adaptive immune response. These exact activities remain to be defined but could include both effector and regulatory functions (60, 61). We suggest that metabolic reprogramming, in particular increased levels of glycolysis, is an essential part of this second wave and that mTORC1 is a critical component of the process. In this model, it is likely that effector functions and metabolic changes are more sensitive to the effects of rapamycin and that metabolism may have a more profound effect on these later stage effector functions. In support of this, direct glycolytic restriction appears to have a greater effect on cytokine-stimulated NK cells that have been activated for longer periods and undergone glycolytic reprogramming compared to short-term stimulated NK cells (14, 15, 24). Understanding how NK cell metabolism is regulated at these longer time periods will be important for our understanding of NK cell dysfunction in chronic diseases. Ultimately, this knowledge will help to refine existing therapies but also provide new opportunities for NK cell-based immunotherapies for the treatment of cancer and chronic viral infections.

## Author Contributions

All authors listed have made substantial, direct, and intellectual contribution to the work and approved it for publication.

## Conflict of Interest Statement

The authors declare that the research was conducted in the absence of any commercial or financial relationships that could be construed as a potential conflict of interest. The reviewer CJ and handling editor declared their shared affiliation, and the handling editor states that the process nevertheless met the standards of a fair and objective review.
